# Structure-based screening of binding affinities via small-angle X-ray scattering

**DOI:** 10.1107/S2052252520004169

**Published:** 2020-05-06

**Authors:** Po-chia Chen, Pawel Masiewicz, Kathryn Perez, Janosch Hennig

**Affiliations:** a Structural and Computational Biology Unit, EMBL Heidelberg, Meyerhofstrasse 1, 69126 Heidelberg, Germany; b Protein Expression and Purification Core Facility, EMBL Heidelberg, Meyerhofstrasse 1, 69126 Heidelberg, Germany

**Keywords:** drug discovery, molecular recognition, solution scattering, structural biology, SAXS

## Abstract

Protein–protein and protein–ligand interactions often involve conformational changes or structural rearrangements that can be quantified by solution small-angle X-ray scattering (SAXS). These scattering intensity measurements reveal structural details of the bound complex, the number of species involved and, additionally, the strength of interactions if carried out as a titration. Although a core part of structural biology workflows, SAXS-based titrations are not commonly used in drug discovery contexts. This is because prior knowledge of expected sample requirements, throughput and prediction accuracy is needed to develop reliable ligand screens. This study presents the use of the histidine-binding protein (26 kDa) and other periplasmic binding proteins to benchmark ligand screen performance. Sample concentrations and exposure times were varied across multiple screening trials at four beamlines to investigate the accuracy and precision of affinity prediction. The volatility ratio between titrated scattering curves and a common apo reference is found to most reliably capture the extent of structural and population changes. This obviates the need to explicitly model scattering intensities of bound complexes, which can be strongly ligand-dependent. Where the dissociation constant is within 10^2^ of the protein concentration and the total exposure times exceed 20 s, the titration protocol presented at 0.5 mg ml^−1^ yields affinities comparable to isothermal titration calorimetry measurements. Estimated throughput ranges between 20 and 100 ligand titrations per day at current synchrotron beamlines, with the limiting step imposed by sample handling and cleaning procedures.

## Abstract 2

A feasibility study on how the binding affinities of protein–ligand interactions are obtained, based on the solution scattering intensities of their respective titration series, is presented. Experimental parameters and their resulting influence on accuracy and precision are analysed.

## 1. Introduction   

**Figure 1:**
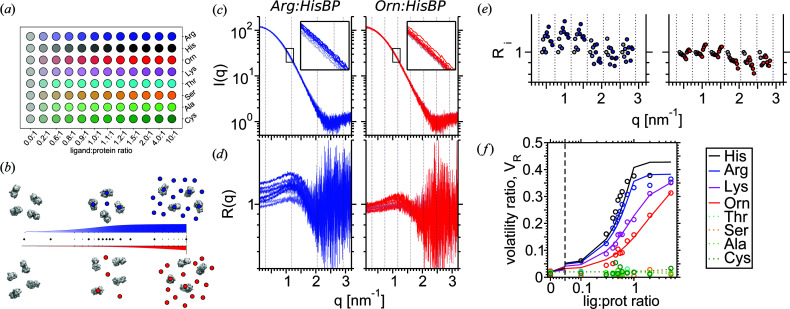
SAXS-based screening using the volatility ratio *V*_R_, exemplified by the histidine-binding protein (HisBP) titrations. (*a*) 96-well plate setup for small-scale screening divided into eight twelve-point titrations, spanning from the *apo* ligand-less measurement to the saturated ligand-excess measurement. (*b*) Theoretical binding curves for a strong (blue) and weak (red) receptor–ligand interaction at constant receptor concentrations. The former reaches saturation at approximately 1:1 ratio, whereas the latter does not saturate even at 10:1 excess. (*c*) Example titrations of 160 µ*M* HisBP against Arg and Orn, visualized by the buffer-subtracted SAXS intensity *I*(*q*). The *apo* *I*(*q*) is coloured grey, with SAXS profiles of other titration points coloured according to ligand:receptor ratios. (*d*) Normalized ratio *R*(*q*) versus *apo* protein *I*(*q*), matching equation (4). (*e*) Geometric average *R*_*i*_ for each complete Shannon channel, matching equation (6). Points have been horizontally shifted to aid visualization. (*f*) Computed *V*_R_ curves for a preliminary titration of HisBP versus eight active and non-active amino-acids.

Small-angle X-ray scattering (SAXS) is a widely used technique to examine structural features on the micrometre and nanometre scales, offering ready access to the physical behaviours of biomolecules in the solution environment (Svergun *et al.*, 2013 ▸; Putnam *et al.*, 2007 ▸; Skou *et al.*, 2014 ▸). Within this context, SAXS reports the globally averaged distance distribution between scattering electron densities around all atoms. This distribution is obtained via measuring the excess intensity of a sample solution over that of an equivalent buffer solution without sample. The scattered photon intensity *I*(*q*) as a function of the momentum transfer *q* and its associated scattering angle 2θ between the incident and deflected beams obeys 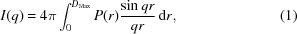


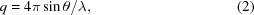
where *P*(*r*) is the desired distance distribution between all atoms and in the nearby molecule with maximum spatial diameter *D*_max_, weighted by the excess electron density. The globally averaged properties of SAXS imply that a mixture of multiple species with negligible long-range spatial interactions will linearly contribute their respective scattering intensities. Thus, by titrating two species at different input concentrations and measuring *I*(*q*) at each point, their populations at equilibrium can, in principle, be retrieved along with respective structural information. This is the basis of SAXS-based ligand screening (Tuukkanen & Svergun, 2014 ▸; Chen & Hennig, 2018 ▸). To illustrate this, consider a simple two-state interaction between a receptor *R* and its ligand *L* which forms the complex *RL*. The concentrations of these three species at equilibrium are governed by a dissociation constant *K*_D_ which describes the strength of the interaction, 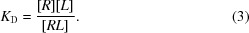
Thus, *K*_D_ can be computed by titrating *R* and *L* at multiple input ligand:receptor ratios, then deriving the solution concentrations of [*R*], [*L*] and [*RL*] under the assumption that each is distinguishable in SAXS. In the general case, the SAXS profiles of all three species must be modelled. However, the scattering contributions of small molecules are generally much weaker than receptor scattering, and are almost constant over the *q*-ranges covered by SAXS. In such cases, the contribution of a ligand *L* can be modelled by constant scattering and removed by numerical fitting, as commonly carried out for buffer matching (Petoukhov *et al.*, 2012 ▸; Schneidman-Duhovny *et al.*, 2013 ▸; Chen & Hub, 2014 ▸). If one assumes that the remaining *I*(*q*) changes directly correspond to the balance between *R* and *RL* after constant contribution, then dissociation constants can be directly determined from perturbations of the SAXS signal (Fig. 1 ▸).

The availability of high-intensity synchrotron sources and automated workflows enables precise SAXS measurements within seconds (Classen *et al.*, 2013 ▸; Kirby *et al.*, 2013 ▸; Acerbo *et al.*, 2015 ▸; Blanchet *et al.*, 2015 ▸), or far less using time-resolved setups (Cammarata *et al.*, 2008 ▸). Thus, in the context of screening, a single synchrotron experiment can feasibly screen a small set of candidate ligands and identify members that interact strongly, weakly or not at all. The precision is such that SAXS can track subtle changes regardless of whether the biomolecules are composed of well folded domains (Rambo & Tainer, 2013*b* ▸; Vestergaard & Sayers, 2014 ▸) or disordered chains (Kikhney & Svergun, 2015 ▸; Cordeiro *et al.*, 2017*b* ▸). This capability has been leveraged to conduct titrations of diverse biomolecular systems: proteins (Ando *et al.*, 2012 ▸, 2016 ▸; Meisburger *et al.*, 2016 ▸; Cordeiro *et al.*, 2017*a* ▸; Fukuda *et al.*, 2017 ▸; Tian *et al.*, 2014 ▸; Herranz-Trillo *et al.*, 2017 ▸; Chen *et al.*, 2018 ▸), nucleic acids (Meisburger *et al.*, 2013 ▸; Chen & Pollack, 2016 ▸), detergents (Lipfert *et al.*, 2007 ▸; Oliver *et al.*, 2013 ▸) and others, in order to expose possible structural mechanisms that underlie their functional behaviour.

The majority of the above studies concentrate on the structural information obtained, although several also utilize SAXS to additionally compute *K*_D_ (Cordeiro *et al.*, 2017*a* ▸) or derive IC_50_ estimates (Meisburger *et al.*, 2016 ▸; Ando *et al.*, 2012 ▸, 2016 ▸). Broad utilization of affinity-based applications is hindered by a number of practical difficulties. Firstly, a ligand screen may potentially produce novel complex conformations that are not known prior to the experiment. This complicates the application of population modelling to compute *K*_D_ directly, since the scattering signals of the corresponding purified complex are not available. A reliable alternative procedure for *K*_D_ predictions must be found using a replacement metric. Secondly, no broadly applicable guidelines have yet been established on how to formulate protocols for SAXS-based ligand screening. Although prospective beamline measurements will provide sufficient context to establish sample requirements and measurement protocols, one lacks screening-specific details such as their influence on the observable range and precision of *K*_D_ values. To help tackle this challenge, the histidine-binding protein (HisBP) is selected here as a standard reference for ligand screening, on the basis of its membership in the periplasmic binding protein family (PBP). PBPs serve as *in vivo* nutrient sensing and transport proteins (Tam & Saier, 1993 ▸; Quiocho & Ledvina, 1996 ▸) and offer advantages of relative stability plus SAXS-detectable compaction of its two-lobed domain upon ligand capture (Olah *et al.*, 1993 ▸). The results below will first cover the performance of HisBP screens versus nanomolar to micromolar ligands at a single beamline, followed by effects of exposure protocols on screening performance at multiple beamlines. Additional protocol validation using two other PBP members glutamine (GlnBP) and aspartate/glutamate (DEBP) binding protein will be included. These investigations establish preliminary guidelines on effective screening, and suggest that the volatility ratio *V*_R_ is a suitable proxy metric to extract underlying populations from two-state mixtures, enabling the possibility of generalized ligand screening without explicit modelling of scattering curves.

## 2. Theory   

### 2.1. Quantifying the difference between scattering curves by volatility ratios   

According to Shannon channel information theory, any SAXS profile can be captured by a small number of independent parameters. Similarly, one can also summarize the difference between any two scattering profiles *I*(*q*) and *J*(*q*) by changes in related structural parameters such as the radius of gyration *R*_g_, Porod volume *V*_P_ and volume of correlation *V*_c_ (Rambo & Tainer, 2013*a* ▸). Alternatively, explicit difference measures such as linearity of fit, χ_lin._ (Chen *et al.*, 2018 ▸), and volatility ratio *V*_R_ (Hura *et al.*, 2013 ▸) can be computed. Each of these quantities smoothly captures the net change in scattering profiles due to changes in the underlying populations of the bound and unbound receptor. In particular, *V*_R_ quantifies SAXS differences by computing the ratio of intensities between the two curves and summing the scalar changes of mean ratios between adjacent Shannon channels in *q*-space. To give an analogy, the *V*_R_ metric is sourced from the field of economics where it describes which of two stock prices has exhibited more rapid fluctuations over a period of time.

A modified definition of *V*_R_ is presented below, adapted for ligand-based screening. For a given set of titrations conducted at the same receptor concentration and beamline session, a reference profile *J*(*q*) is defined by averaging all buffer-subtracted scattering profiles measured on the apo protein. The geometrically normalized ratio *R*(*q*) between *J*(*q*) and *I*(*q*) at each titration point is evaluated, ignoring any points where *I*(*q*) < 0 or *J*(*q*) < 0: 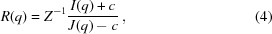


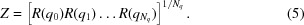
Figs. 1 ▸(*c*) and 1(*d*) illustrate the *I*(*q*) and *R*(*q*) series of two ligand titrations Arg:HisBP and Orn:HisBP, defining *J*(*q*) as the respective apo intensities. Here, *N*_*q*_ defines the total number of raw measurements over scattering angles *q*, such that *N*_*q*_ is much greater than the number of associated independent Shannon channels *N*_S_. An optional fitting constant *c* is applied to take into account variations in buffer scattering that arise from unbound ligands and counterions, while preserving the symmetry property *V*_R_.

To account for the oversampling of SAXS profiles relative to the Shannon information content, *R*(*q*) is divided along *q* into *N*_S_ channels of width Δ*q* = π/*D*_max_, resulting in *M* channels and *M*(*q*) points within each complete channel. A fixed *D*_max_ is chosen based on the largest observed particle size across the titration. Applying geometric means again produces a mean ratio *R*_*i*_ for each channel [Fig. 1 ▸(*e*)], 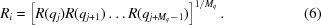
In practice, the usable *q*-range is rarely an integer multiple of Shannon channels. A weight factor *w_i_* is thus applied, defined to be 1 for all channels except for the partial bin at the highest *q*, where the number of raw measurements in the channel *N*_*q*,channel_ may be less than *M*_*q*_. *V*_R_ is defined as the sum of scalar differences between consecutive *R*_*i*_,
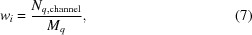


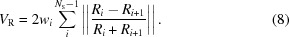
In this study, *V*_R_ is numerically minimized over the constant parameter *c* by Powell minimization (Powell, 1964 ▸), with its initial value set to zero.

## 3. Materials and methods   

### 3.1. Sample preparation   

The *E. coli* HisBP gene (Uniprot entry P0AEU0, residues 23–260) was cloned into pET-M11 plasmids containing kanamycin resistance, T7-lac promoter and an N-terminal His_6_ tag with a TEV cleavage site. The pETMSCIII plasmids for GlnBP (Uniprot entry P0AEQ3, residues 23–248) and DEBP (Uniprot entry P37902, residues 28–302) were generously provided by Professor Colin Jackson, containing ampicillin resistance and N-terminal His_6_ tags.

The production and purification of GlnBP and DEBP follow that of previous protocols (Clifton & Jackson, 2016 ▸). We summarize this below with slight modifications for HisBP. After plasmid amplification in *E. coli* DH5α cells, protein expression was carried out in *E. coli* BL21(DE3) cells. Expression cultures were grown in LB medium at 37°C supplemented with either 100 µg ml^−1^ ampicillin (GlnBP/DEBP) or 50 µg ml^−1^ kanamycin (HisBP). For NMR experiments, LB medium was replaced by ^15^N-labelled M9 minimal medium. Once OD_600_ exceeded 0.6, cultures were induced with 1 m*M* isopropyl β-d-1-thiogalactopyranoside for 4 h. Cells were spun-down, resuspended in loading buffer (500 m*M* NaCl, 20 m*M* imidazole and 20 m*M* NaH_2_PO_4_ pH 7.4), lysed by sonication, then filtered before conducting His_6_-tag-based purification by Ni-nitrilotriacetic acid affinity (Ni-NTA) chromatography. After initial loading and washing, on-column refolding was conducted via a decreasing urea gradient from 8 *M* to 0 *M* to remove bound endogenous ligands, then eluted in buffer (500 m*M* NaCl, 300 m*M* imidazole and 20 m*M* NaH_2_PO_4_ pH 7.4). HisBP was further exchanged back into the loading buffer, cleaved overnight via addition of TEV protease, then re-loaded onto a second Ni-NTA column to separate the similar-sized N-terminal tags. All PBPs were finally subjected to size-exclusion chromatography (SEC) in the final buffer used for all experimental measurements (100 m*M* NaCl, 0.5 m*M* TCEP and 20 m*M* NaH_2_PO_4_ pH 7.4), except for NMR meaurements where samples were diluted by the addition of 9% D_2_O.

### 3.2. SAXS measurements   

Candidate ligands for each protein were selected on the basis of known affinities in the literature and chemical similarity. Titrations were conducted at fixed protein concentrations to exclude signal-to-noise factors. An initial mass concentration of 2.0 or 4.0 mg ml^−1^ was employed in prospective experiments, then scaled to 2.0, 1.0, 0.5 and 0.25 mg ml^−1^ in later concentration-variation studies. Twelve-point titrations were prepared at ligand-protein ratios 0.0, 0.2, 0.6, 0.8, 0.9, 1.0, 1.1, 1.2, 1.5, 2.0, 4.0 and 10.0 to cover a theoretical *K*_D_ sensitivity within two orders of magnitude of the fixed protein concentration employed, assuming ∼5% random error (Fig. S1 of the supporting information). A total of eight beamline experiments were conducted, using the automated sample changing environments available on site, operated in constant-flow mode: three times at ESRF BM29, three at DESY P12, once at Australian Synchrotron SAXS/WAXS and once at Diamond B21. Of the above, one prospective session at ESRF and two at DESY were dedicated to identifying a suitable set of PBP interactions and optimizing protocols. Their preliminary results have been excluded from this report. Furthermore, experiments at ESRF BM29 and Diamond B21 were carried out on site, while the DESY P12 experiment was carried out by mail-in. For these measurements, samples were prepared by pipetting ligand–protein mixtures onto 96-well plates prior to transport. As for the Australian Synchrotron measurements, apo–HisBP was lyophilized in its final buffer and reconstituted on site using pure H_2_O and then mixed with the ligands in 96-well plates. The measurement parameters for each site are catalogued in Table 1 ▸. For brevity, we report six representative scattering curves for HisBP, GlnBP and DEBP in their free and native ligand-bound forms in Table S5 of the supporting information following community guidelines (Trewhella *et al.*, 2017 ▸).

### 3.3. Automated analysis pipeline   

The *SAXScreen* workflow was utilized for the semi-automated prediction of binding curves from scattering intensities (Chen *et al.*, 2018 ▸), whose methodology we reproduce here with modifications for quantitative prediction. Buffer-subtracted intensities provided from respective synchrotron pipelines were used as starting points for analysis. *DATGNOM* (Franke *et al.*, 2017 ▸) was used to determine the maximum molecular extent, *D*_max_, for each PBP, which ranges from 5.6 to 8.2 nm depending on the protein size and ligand binding (*cf.* Table  S5). The average values for apo–HisBP, GlnBP and DEBP were determined to be 5.9 ± 0.1, 6.4 ± 0.3 and 8.2 ± 0.2 nm, respectively. Thus, an intermediate value of 7.2 nm was fixed for all computations involving *D*_max_ including *V*_R_.

*ATSAS AUTORG* (Petoukhov *et al.*, 2007 ▸) was utilized to algorithmically compute scattering angle regions obeying the Guinier approximation (Fig. S2). This was used to assist in locating data points affected by sample handling errors or measurement failures, along with detectable aggregation. The ideal minimum angle after removal of parasitic scattering is 0.1–0.15 nm^−1^, although a more conservative cutoff was used to collectively eliminate residual aggregation. The maximum usable *q* was determined by visual inspection of noise. The final ranges used across all HisBP beamline sessions were: 0.2–2.0 nm^−1^ at 20 µ*M* and below, 0.25–2.5 nm^−1^ at 40 µ*M*, and 0.3–3 nm^−1^ above 80 µ*M*. The *q*-ranges for DEBP and GlnBP were set to 0.1–2.8 nm^−1^.

We considered the data-cleaning algorithms provided by *SAXS Merge* to further exclude user bias (Spill *et al.*, 2014 ▸), but did not use this in the final analysis. A common scattering reference is created per HisBP concentration and per beamline experiment by averaging apo protein replicates after discarding outliers. This reference is used for both *V*_R_ and χ_lin_ computations. Other metrics have been computed as previously reported (Chen *et al.*, 2018 ▸).

**Figure 2:**
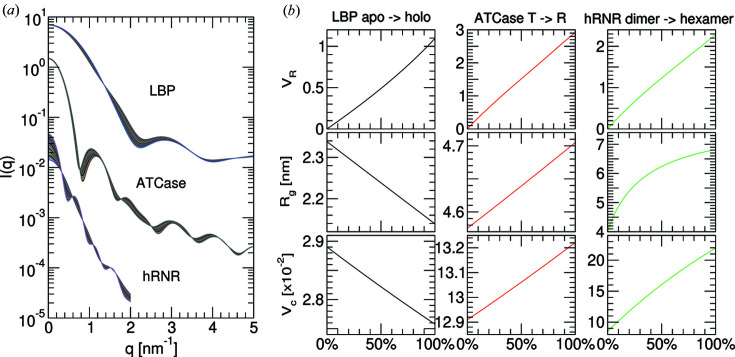
Theoretical modelling of the changes in structural parameters as a function of the mixtures of two conformers. (*a*) Theoretical SAXS profiles of three ligand-triggered population transitions, following open versus closed leucine-binding protein (LBP), *T* versus *R* states of aspartate carbamoyltransferase (ATCase) and dimeric versus hexameric forms of human ribonucleotide reductase (hRNR). Profiles were computed by *CRYSOL* based on crystal structure coordinates, see the main text for details. (*b*) Corresponding structural parameters computed from a mixture of two conformations for *V*_R_, *R*_g_ and *V*_c_. *V*_R_ values were calculated over 0–2 nm^−1^ for LBP and ATCase, and over 0–1 nm^−1^ for hRNR.

To fit binding curves against metrics, we consider all titrations conducted per protein concentration per beamline experiment as a single set, after removing data points resulting from aggregation, preparation errors or measurement errors. For a given metric *X*, model curves are constructed using the following free parameters: the expected value for the apo receptor *X*_apo_, a set of values for each receptor–ligand *X*_holo_(*L*) and a set of ligand affinities log_10_ *K*_D_(*L*). As the majority of metrics vary pseudo-linearly with population changes (Fig. 2 ▸), we adopt the following linear model for simplicity,
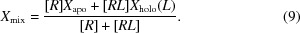
The set of model curves together with equation (3) were fitted against observed data using a Powell minimization algorithm (Powell, 1964 ▸). Since the two-state binding curve at constant [*R*] cannot effectively discriminate between *K*_D_ values much larger or smaller than [*R*], fitted *K*_D_ values are limited to within four orders of magnitude of the receptor concentration (*cf.* Fig. S1). Two uncertainty estimation methods were implemented to define error bars in *K*_D_. For metrics possessing measurement uncertainty (χ_lin_, *R*_g_ and *V*_c_), 1000 replicates were produced by adding Gaussian noise with σ equal to the measurement uncertainty. Error bars indicate σ of log*K*_D_ from the ensemble of replicate predictions. For metrics where uncertainty was not computed (*V*_R_ and *V*_P_), the uncertainty was estimated by single-point removal, where *K*_D_ was recomputed after removal of each titration point. The σ of the resulting replicates are taken as the σ of log*K*_D_. To eliminate cases where the fitting process fails due to ill-constrained parameters, replicates were discarded if the fitted apo–holo differences were >3σ from the mean value of all replicates.

### 3.4. Isothermal calorimetry   

All ITC measurements were conducted on a Malvern MicroCal PEAQ ITC at 20°C with stirring at 200 rev min^−1^ using the same buffer as in SAXS measurements. The cell was initially loaded with 200 µl of 20 µ*M* protein with an initial ligand concentration of 200 µ*M* in the syringe to quantify His:HisBP binding. This was further adjusted in subsequent runs to resolve weaker interactions as necessary. ITC titration curves for all experiments are shown in Fig. S3 along with the concentrations used.

### 3.5. NMR   

All NMR experiments have been carried out at 298 K on an 800 MHz Bruker Avance III NMR spectrometer equipped with a cryogenic probehead. Titrations of HisBP against His and Arg were measured via recording ^1^H–^15^N HSQC spectra at discrete ligand:protein ratios of 0.0, 0.1, 0.2, 0.3, 0.4, 0.6, 0.8, 0.9, 1.0, 1.1, 1.2 and 1.5 for His; and 0.0, 0.1, 0.2, 0.3, 0.4, 0.6, 0.8, 0.9, 1.0, 1.1, 1.2, 1.5, 1.8 and 2.1 for Arg, respectively. Data were processed with *NMRpipe* (Delaglio *et al.*, 1995 ▸), analyzed using *NMRFAM-Sparky* (Lee *et al.*, 2015 ▸) and visualized with Python modules *nmrglue* and *matplotlib* (Helmus & Jaroniec, 2013 ▸; Hunter, 2007 ▸).

Backbone assignments of apo- and His-bound HisBP have been derived from BMRB entries 19242 and 19245 (Chu *et al.*, 2013 ▸). The assignment of Arg-bound HisBP is produced by extending the apo-HisBP assignment along titrations, leaving sites in slow exchange unassigned. For affinity computations based on chemical shift perturbations, ^1^H and ^15^N shifts have been combined to a composite shift δ = [(δ_H_)^2^ + (0.2δ_N_)^2^]^1/2^. The derivation of an overall dissociation constant from chemical shift perturbations follows the work by Williamson (2013 ▸), where the shift in peak position is used to derive free and bound populations without considering peak intensities. The effect of site-specific exchange rates upon peak positions is taken into account (London, 1993 ▸). We discarded residues with either insignificant shifts at saturation (δ < 0.05) or which were in slow exchange such that peak positions alone were no longer informative. This resulted in 110 sites included in the fitting process. The binding interaction is modelled with site-specific bound-state CSP, site-specific bound-state lifetimes τ and a single overall *K*_D_. This translates to 221 degrees of freedom, fitted to 1468 target CSP values.

### 3.6. Software and data availability   

The *SAXScreen* software has been previously published at https://www.github.com/zharmad/SAXScreen and has been updated to include *V*_R_ computations. The dataset of the 1D sample and buffer scattering intensities for each beamline has been made available on Zenodo and accessible via https://dx.doi.org/10.5281/zenodo.3355877. Representative scattering datasets have been deposited at SASBDB (Valentini *et al.*, 2015 ▸), encompassing HisBP, DEBP and GlnBP in their unbound states and bound to eponymous cognate ligands (SASBDB identifiers: SASDFD8, SASDFE8, SASDFF8, SASDFG8, SASDFH8 and SASDFJ8).

## 4. Results   

### 4.1. Theoretical modelling   

To enumerate the relationship between the populations of two-state mixtures and the metrics computed from their composite scattering profiles, theoretical modelling was conducted to simulate three ligand-triggered structural transitions, utilizing six atomic coordinates from the PDB: the closure of leucine-binding protein (LBP) upon ligand capture by 1usg and 1usi (Magnusson *et al.*, 2004 ▸), the product-regulated switching between a low activity T-state and a high-activity R-state of aspartate carbamoyltransferase (ATCase) by 1za1 and 7at1 (Wang *et al.*, 2005 ▸; Gouaux *et al.*, 1990 ▸), and the assembly of dimeric versus hexameric states of human ribonucleotide reductase (hRNR) by 3hnc and 6aui (Fairman *et al.*, 2011 ▸; Brignole *et al.*, 2018 ▸). Fig. 2 ▸ demonstrates that, assuming ligand contributions have been subtracted, the change in population produces quasi-linear changes in the structural metrics. The effective *R*_g_ versus population curve is strongly non-linear for the dimer–hexamer transition of hRNR due to the non-monodispersity of intermediate mixtures. In contrast, *V*_R_ and *V*_c_ deviate by <3% from linear regression fits across all three theoretical titrations. This suggests that populations can be directly estimated from a linear fit to parameter changes. A more accurate polynomial fit appears to be non-trivial to devise, as the curvature depends on the scattering molecules.

### 4.2. HisBP screening at Diamond B21   

**Figure 3:**
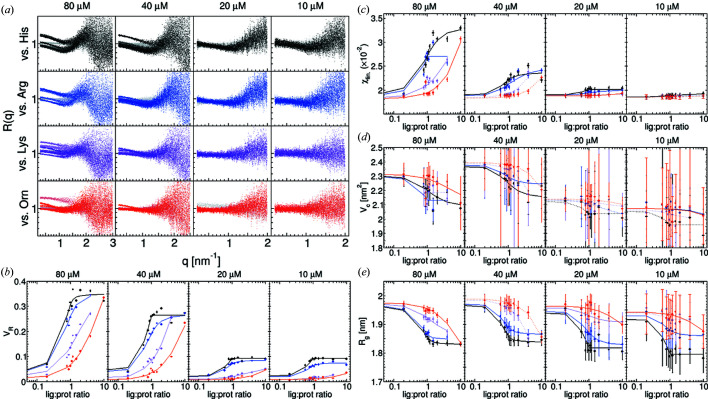
Detailed analysis of HisBP ligand screening at Diamond B21 at four concentrations between 10 and 80 µ*M*. Colours represent HisBP titrated against four ligands: His (black), Arg (blue), Lys (violet) and Orn (red). (*a*) *R*(*q*) plotted over the *q*-range included in the analysis. (*b*) Computed *V*_R_ curves. (*c*) Computed χ_lin_ curves. (*d*) Computed *V*_c_ curves. (*e*) Computed *R*_g_ curves.

The 26 kDa HisBP possesses four known amino acid ligands with *K*_D_ ranging from 64 n*M* to 72 µ*M*: histidine, arginine, lysine and ornithine (Table 2 ▸). Titrations against each ligand were performed at 10, 20, 40 and 80 µ*M* at a total photon dose of 10^14^ to evaluate the impact of receptor concentrations on screening performance (Fig. 3 ▸). First, both the signal-to-noise ratio (S/N) and usable *q*-range were found to be dependent on the receptor concentration employed, as would be expected when the total dose is held constant. In the case of HisBP, dropping concentrations from 40 µ*M* to 20 µ*M* leads to loss of detectable intensity changes above *q* > 2  nm^−1^ (*cf.* Fig. S4). However, their effects upon structural parameters are different: the comparative parameters *V*_R_ and χ_lin_ decrease in magnitude with decreasing concentration, but their uncertainties remain constant. Even at the minimum 10 µ*M* HisBP, *V*_R_ binding curves could still be resolved. In contrast, the structural parameters *V*_c_ and *R*_g_ increase in uncertainty with decreasing concentration. This increase in uncertainty is larger than the total observed change caused by ligand binding at 10 and 20 µ*M*, although the size of the errors is likely to be over-estimated.

### 4.3. Replication with varying photon doses   

**Figure 4:**
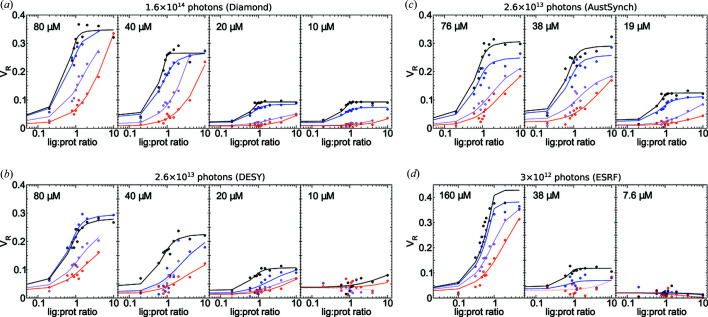
Replicate *V*_R_ titration curves of HisBP against four ligands: His (black), Arg (blue), Lys (violet) and Orn (red). Data collected at various total doses: (*a*) 1.6 × 10^14^ photons at Diamond, *N* = 192; (*b*) 2.6 × 10^13^ photons at DESY, *N* = 172; (*c*) 2.6 × 10^13^ photons at Australian Synchrotron, *N* = 144; and (*d*) 3 × 10^12^ photons at ESRF, *N* = 144. The HisBP concentrations used within each experiment are labelled on respective plots. Note that the maximum *q*-range measured in (*d*) was 2.8 nm^−1^, which affected the *V*_R_.

The accuracy of the above metrics and final uncertainties are affected by the S/N of the measured SAXS profiles. Thus, replicate HisBP screening trials were carried out at three other beamlines, ESRF BM29, DESY P12 and Australian Synchrotron SAXS/WAXS, so as to gather sufficient data for an analysis based on photon exposure. The summary results of these replicated screening trials are shown in Fig. 4 ▸ and Table 2 ▸, including again HisBP titrations at multiple protein concentrations. For completeness, the binding curves for all parameters and all beamlines are included in Figs. S5–S7.

We first observe that the total photon dose imposes a minimum viable concentration: although 10^14^ photons (Diamond) proves sufficient to permit screening at 10 µ*M* protein (0.26 mg ml^−1^), the use of 10^12^–10^13^ photons at other beamlines resulted in insufficient S/N at this concentration to resolve scattering changes above the noise. In particular, the inter-sample variations increase with a decreasing photon dose. This implies that binding can only be detected in practice if it exerts a *V*_R_ change of >0.1 within the usable *q*-range. The Australian Synchrotron replicate additionally exhibits smoother fits relative to the DESY replicate at the same total dose. This can probably be explained by the division of the total dose into fewer frames at the former, since S/N scales proportionally with photon counts per frame but only as a square root of the number of frames. However, the division of photon exposure should also be balanced with the potential need to remove later frames due to cumulative radiation damage. Dosage comparisons between beamlines are also subject to differences in measuring environment, camera size and other factors influencing the raw counts seen by the detector.

Further analysis of the scattering parameters also suggests a dependence of the maximum *V*_R_ upon ligand identity. The magnitude of the change in *V*_R_ for the native His ligand is consistently but marginally larger than that observed for other binding ligands. This difference is more visible with χ_lin_ binding curves (Fig. S5), further suggesting structural differences between native and non-native complexes. This was confirmed by NMR chemical shift perturbations of HisPB:His and Arg:HisBP, where the two interactions share some but not all contacts (Figs. S8–S10). In particular, the signals from numerous locations around the active site of Arg:HisBP differ from those of His:HisBP, suggesting that the larger Arg side chain enforces an alternate arrangement of the complex.

### 4.4. Dissociation constants from SAXS titrations   

**Figure 5:**
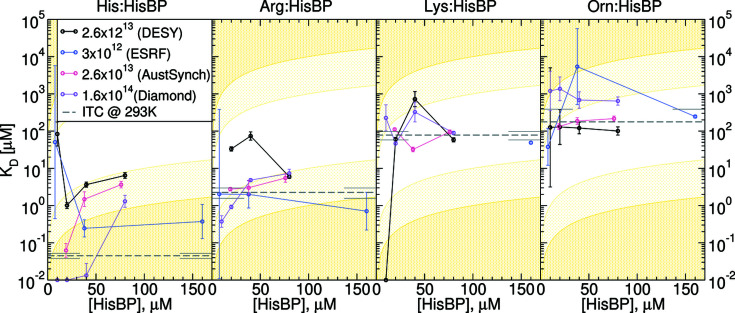
Computed *K*_D_ from *V*_R_ curves in Fig. 4 ▸, plotted as a function of receptor concentration [HisBP] and total photon dose. Colours represent replicates at different total doses: (black) 2.6 × 10^13^ photons at DESY, (blue) 3 × 10^12^ photons at ESRF, (red) 2.6 × 10^13^photons at Australian Synchrotron and (violet) 1.6 × 10^14^ photons at Diamond. Reference values from ITC are shown as dashed grey lines with error bars at the plot edges. Low confidence limits imposed by constant-receptor titrations are represented by light-yellow regions where *K*_D_ lies outside one or two orders of magnitude of [HisBP]. For comparison, equivalent plots using other metrics are shown in Figs. S11–S13.

Dissociation constants (*K*_D_) were computed from *V*_R_ curves by assuming that these accurately represent underlying population changes (Table 2 ▸), with equivalent calculations via other metrics included in Tables S1–S4. Of the Diamond B21 results, the 20 µ*M* titrations appear to be the most consistent with reference values derived from ITC. The largest deviations occur when the target *K*_D_ falls outside two orders of magnitude of receptor concentration (Fig. 5 ▸), or falls below the minimum viable screening concentration. Specifically, the pairing of micromolar [HisBP] required for SAXS and nanomolar His:HisBP *K*_D_ results in a dependence of fitted *K*_D_ values upon [HisBP], which is echoed to a lesser extent by Arg:HisBP. This disparity between SAXS- and ITC-based *K*_D_ appears to be mitigated with higher photon doses, such that the 10^14^ photon dosage utilized at Diamond reliably produced lower nanomolar predictions for His:HisBP. Other residual variations between beamlines arise from sample handling limitations. *AUTORG* analysis of Guinier regimes (Fig. S2) identified a systematic residual aggregation resulting from lyophilization and on-site reconstitution protocols, which affected the Australian Synchrotron measurement. Sporadic sample handling errors and measurement failures also reduced the number of titration points used to determine binding. Notwithstanding these complications, *K*_D_ predictions from *V*_R_ appear to be superior in accuracy to those based on other parameters, which sometimes failed to achieve qualitative ranking (Tables S1–S4). In summary, these results suggest that a viable screening setup for a system comparable to HisBP in terms of size and magnitude of structural change can be screened at 20 µ*M* using 10^13^–10^14^ photons to achieve acceptable resolution between nanomolar and micromolar binding.

### 4.5. Prospective screening of GlnBP and DEBP   

In general, the minimum S/N required to conduct screening depends on the magnitude of the structural changes of a system relative to its physical size. This is relevant to PBPs as they can exhibit a solution equilibrium between open and closed conformations, regardless of ligand binding (Feng *et al.*, 2016 ▸). Thus, the ability for bound ligands to alter the conformational equilibrium affects the minimum viable concentration and total dose. To illustrate the dependence of screening performance on scattering magnitude, prospective screening trials of glutamine-binding protein (GlnBP) and aspartate/glutamate-binding protein (DEBP) are presented. These together with HisBP represent three scenarios: GlnBP exhibits a marginal shift in open–closed equilibria upon ligand binding, DEBP switches from an open-dominated to closed-dominated state, whereas HisBP presented above shifts from an open–closed mixture to a closed-dominated bound complex. This is reflected in the sharp contrast in the net *R*_g_ decrease between the three PBPs (Table 3 ▸).

**Figure 6:**
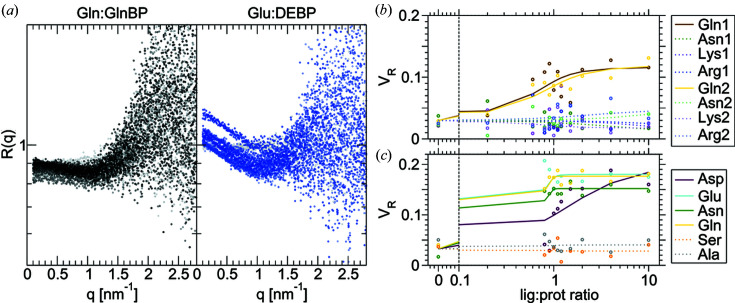
Summary of SAXS-based screening performance on GlnBP and DEBP. (*a*) Ratio curves of *R*(*q*) for the titration between GlnBP and DEBP with its cognate ligands Gln and Glu, respectively. (*b*) *V*_R_ curves for the prospective screening trial of GlnBP with native and other amino acid targets. (*c*) *V*_R_ curves for the prospective screening trial of DEBP with native and other amino acid targets. For comparison, equivalent curves using other metrics are shown in Fig. S14.

Fig. 6 ▸ summarizes GlnBP and DEBP screening conducted at 2 mg ml^−1^ and 10^12^ photons. The small net change of conformations caused by Gln:GlnBP binding translates to miniscule 10% changes in intensities below *q* < 1.5 nm^−1^. This in turn produces poorly defined *V*_*R*_ curves with Δ*V*_*R*_ values of 0.09 versus residuals of ∼0.03. The poor S/N strongly limits resolvable *K*_D_ such that only qualitative binding can be deduced (Table 2 ▸). Notably, the Arg:GlnBP interaction can be detected thermodynamically in ITC, but not structurally in SAXS. This may be due to the Arg side chain stabilizing a complex with dimensions very similar to the average apo distribution. In the same vein, while DEBP experiences a larger compaction on ligand binding relative to HisBP, the compaction is smaller relative to the larger size of DEBP. Thus its Δ*V*_R_ is only 0.16 compared with 0.4–0.5 seen for HisBP. Curiously, the SAXS titrations also suggest an unexpected binding between Asn:DEBP and Gln:DEBP. Here, one notes that selectivity of Asp:DEBP versus Glu:DEBP is 1:10 in ITC but 1:400 in SAXS while Δ*V*_R_ is comparable. This suggests that the Asp:DEBP complex is similar in conformation but less stable than the Glu:DEBP complex. The above analyses illustrate a potential application for SAXS to disambiguate ligand targets on the basis of their structural mechanisms.

## 5. Discussion   

SAXS-based screening fulfils the function of a complementary filter to existing high-throughput methods. Where popular screening platforms offer ligand differentiation based on thermodynamic and functional properties, SAXS offers differentiation based on the equilibrium between structurally distinguishable states, similar to surface plasmon resonance-based screening. This provides information on the structure–function relationship of the screened system prior to full characterization, which can be useful to identify agonists versus inhibitors within drug discovery contexts or relevant complex species within structural biology contexts. In these contexts, SAXS possesses a competitive advantage over alternative structural biology tools in terms of ease of use within the solution environment and low sample requirements. Given sufficient beam time and a well designed protocol, it is possible to derive both the structural states and equilibrium constants governing an interaction.

The screening protocols presented here are limited by the minimum effort required to obtain sufficient measurement precision. The sensitivity range for deriving *K*_D_ from a titration of structurally distinct species depends on the beamline setup, source intensity, available beam time as well as system-specific factors including radiation damage, sample stability and the magnitude of structural change relative to system size. These together determine the viable concentration for screening. The manual pipetting used in the present protocols also imposes limits on the consistency and precision of handling numerous unit microlitre operations, which would be improved by switching to automated liquid handling. Given these limitations, we do not expect SAXS-based screening to provide reliable nanomolar sensitivity for systems less than 100 kDa in size. This shifts the niche for SAXS towards the discovery and filtering of initial leads.

While all tested scattering parameters provide qualitatively usable information on the mixture of states during titration, *V*_R_ appears to be the most reliable method to extract the underlying populations and thus a means to determine *K*_D_. The next best χ_lin_ metric corrects for small concentration variations but over-weighs information from low *q*-angles, and like *R*_g_ it is more vulnerable to residual aggregation artefacts relative to *V*_R_. It is worth noting that no assumption is made of the substrate material during analysis. In addition, the Shannon-channel binning step used here is specific to small-angle scattering. Thus, *V*_R_ can potentially quantify structural alterations in other existing applications of SAXS, for instance cellular ultrastructure (Semeraro *et al.*, 2017 ▸), whole-cell morphology (von Gundlach *et al.*, 2016 ▸) and beyond.

The HisBP titrations conducted here are sufficient to provide initial guidelines on viable SAXS-based ligand screening protocols. A 20 µ*M* limit for HisBP translates to a minimum sample requirement of 0.31 mg per titration, competitive with ∼0.24 mg used per ITC run and 2.4 mg used for NMR titration. This is again likely to hold for similarly sized systems exhibiting comparable structural perturbations. The minimum consumption is ultimately determined by the replicate measurements necessary to reliably determine apo, 1:1 and ligand-excess scattering. This is, in turn, determined by the *accuracy* of *V*_R_ measurements versus the expected change due to structural perturbations. We note that *V*_R_ of independent apo measurements constitutes a measure of accuracy, as its ideal value is zero. In contrast, the expected *V*_R_ changes due to binding are system dependent. While the availability of high-resolution models can be used to estimate an expected Δ*V*_R_ for an arbitrary system, further work will be needed to translate this into suggested total dose, beamline setups and protein concentrations for viable screening. This will also help to determine whether *V*_R_ is a universally applicable metric for *K*_D_ computation, both in terms of the type of structural changes and in terms of reaction complexity. It is likely that other metrics prove to be superior in cases where intensity changes are restricted to particular *q*-regions, or where more than two states are involved. We hypothesize that direct population modelling of intensities will persist as the theor­etically optimal choice, followed by singular vector decomposition methods.

The throughput performance of each beamline used here varies between 3.7 h and 6 h per 96-well plate. Although this clearly depends on net exposure times required to achieve the target dose, for most setups the current limiting step is instead the time required to replace samples and clean the measurement capillary. This can be reduced by duplication of capillaries in existing setups. However, we expect the next breakthroughs in both speed and precision to take place on promising microfluidic chip platforms (Watkin *et al.*, 2017 ▸; Pham *et al.*, 2017 ▸; Schwemmer *et al.*, 2016 ▸). Although not employed here, we note that the SIBYLS beamline claims the highest throughput so far at 15 min per plate with unit second exposures (https://bl1231.als.lbl.gov/htsaxs/instructions/htsaxs). Assuming that 20 s exposure times are required to accumulate 1013 photons needed to screen HisBP, this performance decays to 45 min per plate, sufficient to screen 102 ligands in a 24 h session. For reference, our ITC and NMR protocols require 48 h to accumulate 96 measurements. This is significantly slower, but is less constrained by available access.

## 6. Conclusions   

In summary, this work presents a detailed analysis of the accuracy and precision of SAXS-based determination of the structural equilibrium constant *K*_D_, using sample setups that are competitive with secondary screening approaches used to complement high-throughput screening. In comparison with the throughput of pharmacological screening assays (up to 10^6^ compounds), the throughput here is sufficient to validate a selection of initial hits and inform on the structural implications of ligand binding. This translates to discrimination between likely agonists and antagonists, potential oligomerization and other observations that complement existing pharmacological screening methods.

In this way, SAXS-based ligand screening can benefit high-throughput structural biology and drug discovery pipelines, as an independent source of structural information without expensive isotope-labelling required for NMR, or a search of reliable crystallization protocols. Further work will be needed to confirm that *V*_R_ can be used to directly retrieve *K*_D_ regardless of the biological system being used, which will also contribute towards a useful library of reference data to guide future screening efforts. Along with potential feasibility studies using laboratory-based X-ray sources, we may yet see broad utilization of this particular structure-based screening tool.

## Supplementary Material

NMR titration data, ITC titration data and standardised reporting of representative SAXS measurements. DOI: 10.1107/S2052252520004169/tj5029sup1.pdf

1D sample and buffer scattering intensities.: https://dx.doi.org/10.5281/zenodo.3355877

SASBDB reference: apo HisBP, SASDFD8

SASBDB reference: His-bound HisBP, SASDFE8

SASBDB reference: apo GlnBP, SASDFF8

SASBDB reference: Gln-bound GlnBP, SASDFG8

SASBDB reference: apo DEBP, SASDFH8

SASBDB reference: Glu-bound DEBP, SASDFJ8

## Figures and Tables

**Table 1:** Measurement parameters at the four synchrotron beamlines Note that total dose assumes that all frames have been included for averaging.

Parameter	ESRF	DESY	Diamond	AustSynch
Detector setup	Pilatus-2M	Pilatus-6M	Eiger-4M	Pilatus-1M
Wavelength (nm)	0.9919	1.24403	0.99987	1.07812
*q*-Range (nm^−1^)	0.0306–4.9462	0.0171–7.2507	0.0320–4.3982	0.0540–2.8946
Sample-to-detector distance (m)	2.849	3.0	4.014	2.68
Per-frame exposure (s)	0.5	0.1	2.0	1.0
Flux at sample (photons s^−1^)	5.9 × 10^11^	6.5 × 10^12^	4 × 10^12^	1.3 × 10^12^
Total dose per frame (photons)	3.0 × 10^11^	6.5 × 10^11^	8 × 10^12^	1.3 × 10^12^
Frames	10	40	20	20
Total exposure (s)	5	4	40	20
Total dose (photons)	3.0 × 10^12^	2.6 × 10^13^	1.6 × 10^14^	2.6 × 10^13^
Average frame loss	0	0	0	3
Sample volume (µl)	50	50	50	85
Sample temperature (°C)	20	20	20	20

**Table 2:** Summary of fitted dissociation constants *K*_D_ derived from *V*_R_ titration curves, with comparisons against ITC and NMR measurements Literature ITC data for HisBP was sourced from the work by Paul *et al.* (2016 ▸), and ITC data for DEBP and GlnBP were sourced from the work by Clifton & Jackson (2016 ▸). Titrations that produce no significant changes in scattering curves have been denoted as ‘no change’.

				Ligand identity and *K*_D_			
Protein	Source	Dose (photons)	Concentration (µ*M*)	His	Arg	Lys	Orn
HisBP	ITC, literature	–	–	64 ± 10 n*M*	3 ± 1 µ*M*	19.5 ± 6 µ*M*	72 ± 4 µ*M*
	ITC, this work	–	–	48.0 ± 5.2 n*M*	2.06 ± 0.22 µ*M*	62 ± 11 µ*M*	220 ± 37 µ*M*
	NMR	–	200	–	560 n*M*	–	–
	*V*_R_ (Diamond)	1.6 × 10^14^	80	1.3 ± 0.5 µ*M*	7.3 ± 1.9 µ*M*	89 ± 9.6 µ*M*	640 ± 160 µ*M*
	*V*_R_ (Diamond)	1.6 × 10^14^	40	13 ± 11 n*M*	4.8 ± 0.48 µ*M*	320 ± 210 µ*M*	680 ± 360 µ*M*
	*V*_R_ (Diamond)	1.6 × 10^14^	20	5.1 ± 4.1 n*M*	920 ± 110 n*M*	47 ± 3.6 µ*M*	1.4 ± 1.1 m*M*
	*V*_R_ (Diamond)	1.6 × 10^14^	10	1.4 ± 0.92 n*M*	370 ± 140 n*M*	230 ± 200 µ*M*	1.2 ± 1.8 m*M*
	*V*_R_ (ESRF)	3.0 × 10^12^	160	370 ± 470 n*M*	710 ± 1000 n*M*	49 ± 4.1 µ*M*	250 ± 24 µ*M*
	*V*_R_ (DESY)	2.6 × 10^13^	80	6.4 ± 1.1 µ*M*	5.9 ± 0.6 µ*M*	58 ± 7.5 µ*M*	100 ± 26 µ*M*
	*V*_R_ (AustSynch)	2.6 × 10^13^	76	3.6 ± 0.74 µ*M*	5.5 ± 1.4 µ*M*	95 ± 11 µ*M*	220 ± 34 µ*M*
	*V*_R_ (ESRF)	3.0 × 10^12^	38	240 ± 140 n*M*	2 ± 1.9 µ*M*	n.d.	5.4 ± 28 m*M*
	*V*_R_ (DESY)	2.6 × 10^13^	20	1 ± 0.19 µ*M*	33 ± 4.5 µ*M*	61 ± 6.4 µ*M*	130 ± 170 µ*M*
	*V*_R_ (AustSynch)	2.6 × 10^13^	19	62 ± 28 n*M*	2.8 ± 0.31 µ*M*	110 ± 10 µ*M*	130 ± 24 µ*M*
							
				Glu	Asp	Gln	Asn
DEBP	ITC, literature	–	–	334 ± 120 n*M*	1.73 ± 0.49 µ*M*	–	–
	ITC, this work	–	–	141 ± 66 n*M*	1.15 ± 0.18 µ*M*	–	–
	*V*_R_ (ESRF)	3.0 × 10^12^	80	150 ± 150 n*M*	63 ± 34 µ*M*	≪80 n*M*	≪80 n*M*
							
				Gln	Arg		
GlnBP	ITC, literature	–	–	106 ± 65 n*M*	93.8 ± 7.4 µ*M*		
	ITC, this work	–	–	59 ± 37 n*M*	78.9 ± 8.6 µ*M*		
	*V*_R_ (ESRF)	3.0 × 10^12^	80	8.2 ± 7.5 µ*M*	No change		
	*V*_R_ (ESRF)	3.0 × 10^12^	80	18 ± 10 µ*M*	No change		

**Table 3:** Experimental SAXS *R*_g_ of HisBP, GlnBP and DEBP representative profiles, collected for apo and ligand-excess conditions See Table S5 for the full report.

	SAXS *R*_g_ (Å)			
PBP:ligand	Exp. (apo)	Exp. (saturated)	Δ*R*_g_	Mol. mass (kDa)
His:HisBP	19.6 ± 0.7	18.3 ± 1.0	−1.3	26.3
Gln:GlnBP	21.1 ± 1.4	20.3 ± 1.3	−0.7	25.8
Glu:DEBP	23.2 ± 2.1	20.6 ± 1.3	−2.6	32.1
