# Supplementary material for: Structure-based screening of binding affinities via small-angle X-ray scattering
Source: IUCrJ. 2020 May 6;7(Pt 4):644–55. doi: 10.1107/S2052252520004169 (PMC7340254; doi:10.1107/S2052252520004169)
Supplement: Supplementary file 1 [file m-07-00644-sup1.pdf]

IUCrJ

Volume 7 (2020)

Supporting information for article:

Structure-based screening of binding affinities via small-angle X-ray scattering

Po-chia Chen, Pawel Masiewicz, Kathryn Perez and Janosch Hennig

Supplementary Information for: Structure-based screening of binding affinities via small-angle X-ray scattering

Po-chia Chen[1,*], Pawel Masiewicz[1], Kathryn Perez[1], and Janosch Hennig[1,+]

[1]Structural and Computational Biology Unit, EMBL Heidelberg, Meyerhofstrasse 1, 69126 Heidelberg, Germany
[*]pchen@embl.de
[+]janosch.hennig@embl.de

Supplementary Figures

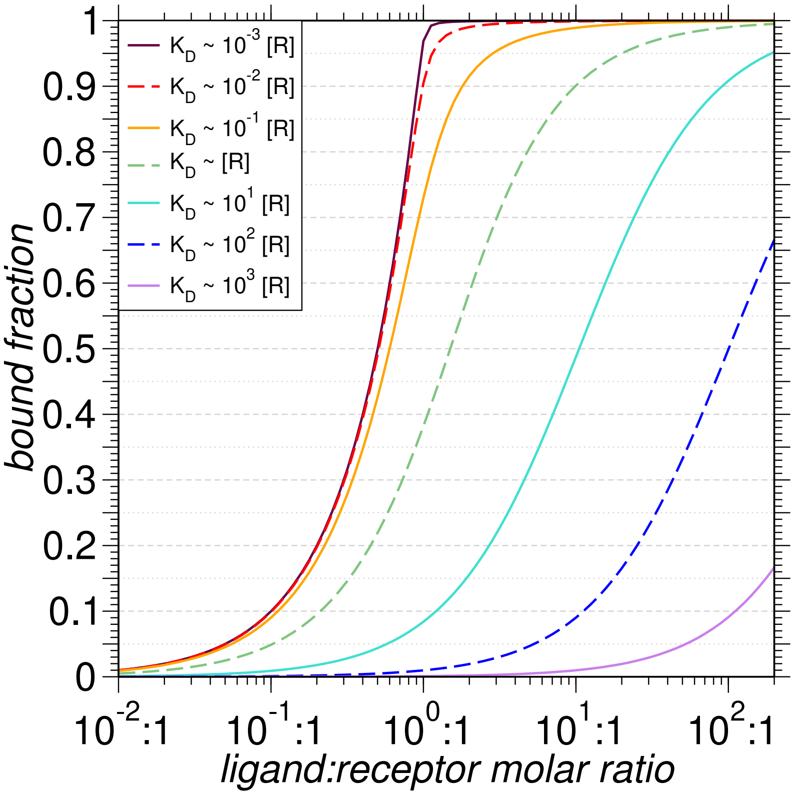

Figure S1. Theoretical two-state binding curve, scaled by the constant receptor concentration.

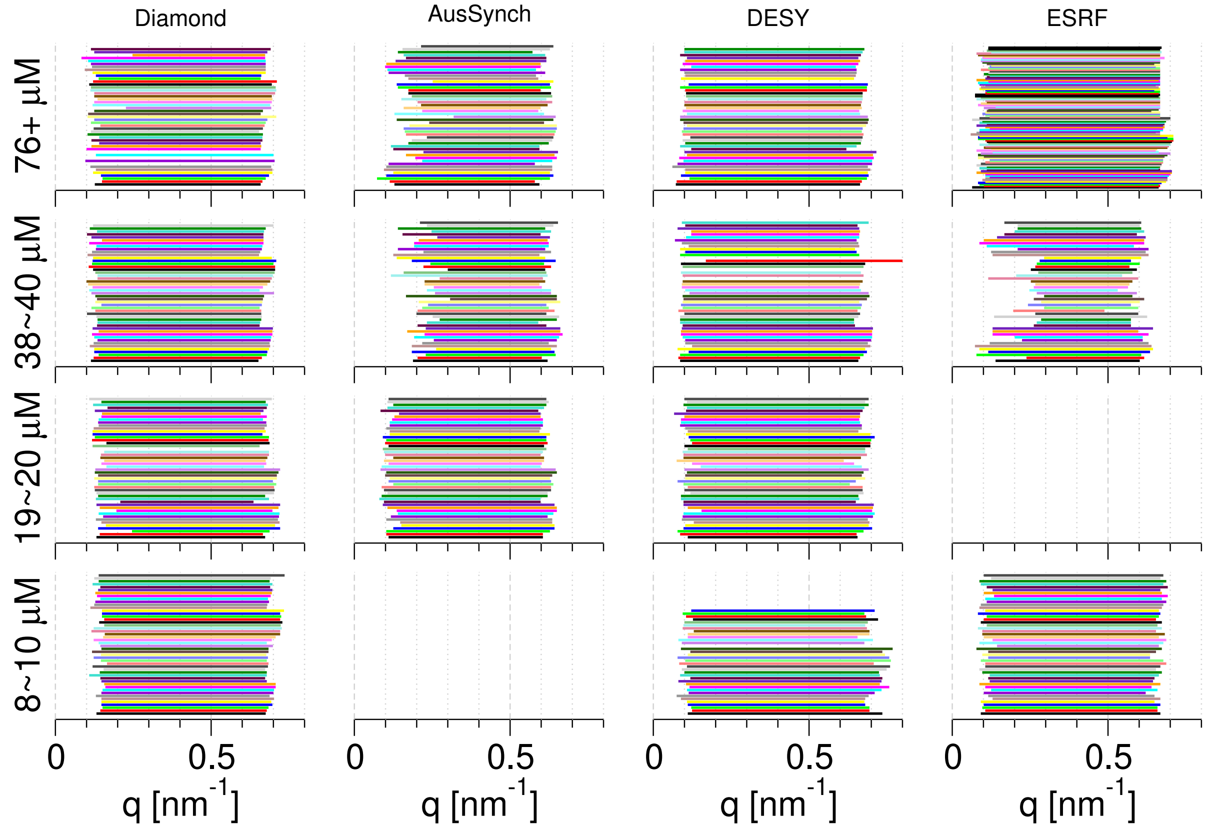

Figure S2. Scattering angles within the Guinier regime for all SAXS measurements reported in this study. Each line represents one buffer-bsubtracted SAXS curve containing sufficient low-angle data to compute a radius of gyration. The range is determined by ATSAS AutoRg, which algorithmically removes data inconsistent with the Guinier approximation $\log I(0) - q^2 R_g^2/3$. Note the sawtooth pattern visible at the maximum Guinier-q, which corresponds to ligand-triggered compaction of HisBP.

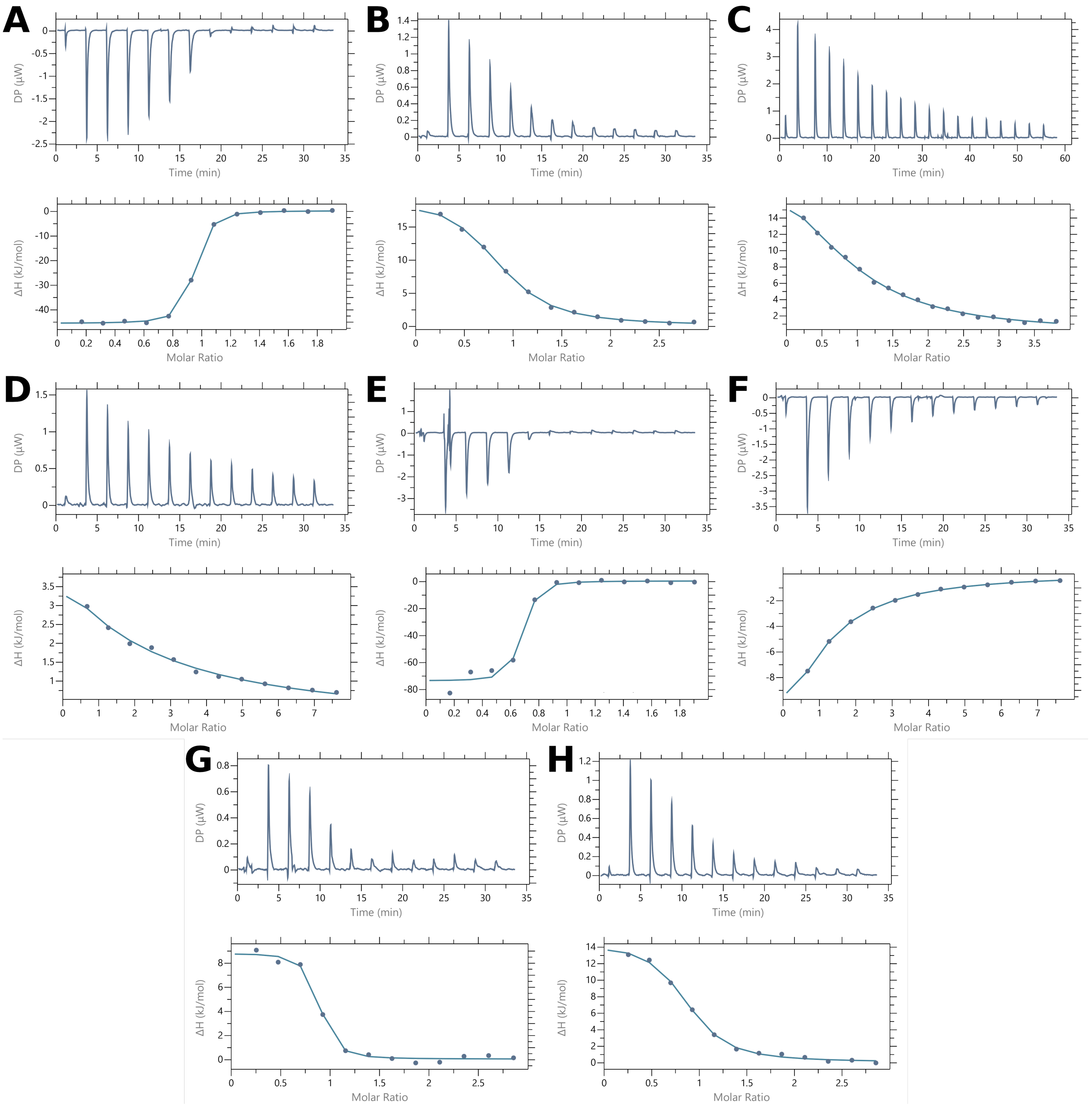

Figure S3. Titration curves of periplasmic binding proteins using isothermal calorimetry: (A) HisBP:His at 20 μM and 200 μM ; (B) HisBP:Arg at 20 μM and 300 μM ; (C) HisBP:Lys at 100 μM and 2 mM ; (D) HisBP:Orn at 50 μM and 2 mM ; (E) GlnBP:Gln at 20 μM and 200 μM ; (F) GlnBP:ARG at 50 μM and 2 mM ; (G) DEBP:Glu at 20 μM and 300 μM ; (H) DEBP:Asp at 20 μM and 300 μM.

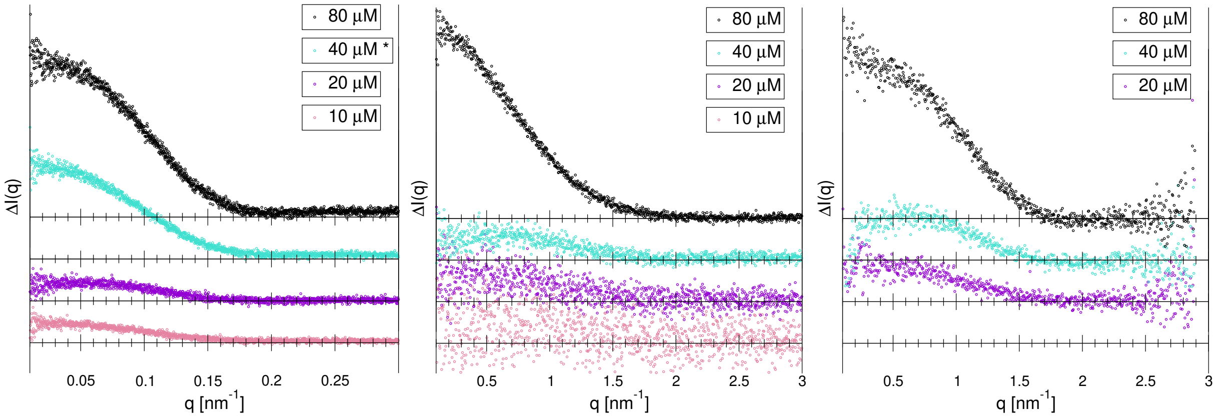

Figure S4. Difference spectra between His-saturated (10:1) and apo HisBP scattering curves as a function of protein concentration. Data collected at Diamond (left), DESY P12 (middle), and Australian Synchrotron SAXS/WAXS (right). The difference curve for His at Diamond, marked with a star, has been replaced with (4:1) measurement.

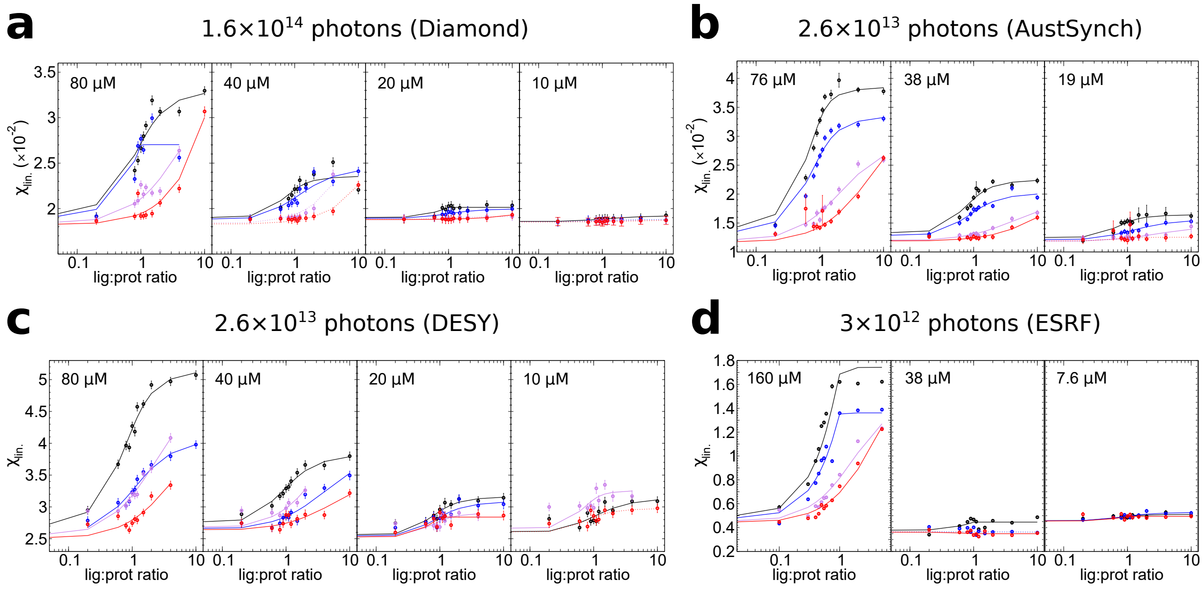

Figure S5. Replicate $\chi_{\mathrm{lin.}}$ titration curves of HisBP against four ligands His (black), Arg (blue), Lys (violet), and Orn (red), at Diamond (a), DESY (b), Australian Synchrotron (c), and ESRF (d). The HisBP concentrations used within each experiment are labelled on respective plots.

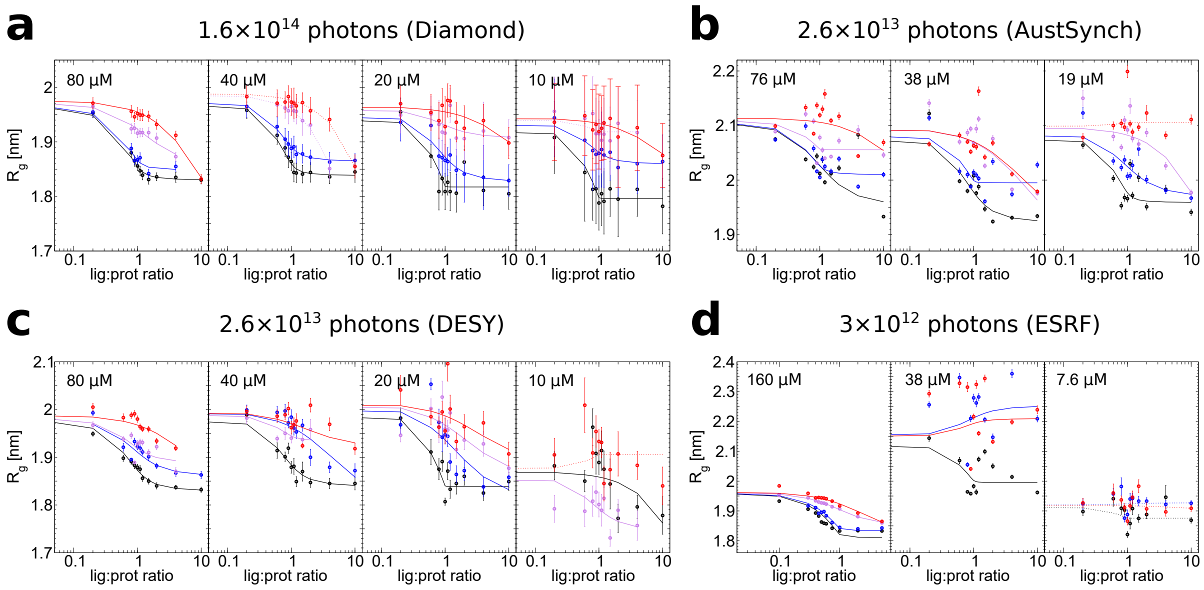

Figure S6. Replicate R_g titration curves of HisBP against four ligands His (black), Arg (blue), Lys (violet), and Orn (red), at Diamond (a), DESY (b), Australian Synchrotron (c), and ESRF (d). The HisBP concentrations used within each experiment are labelled on respective plots.

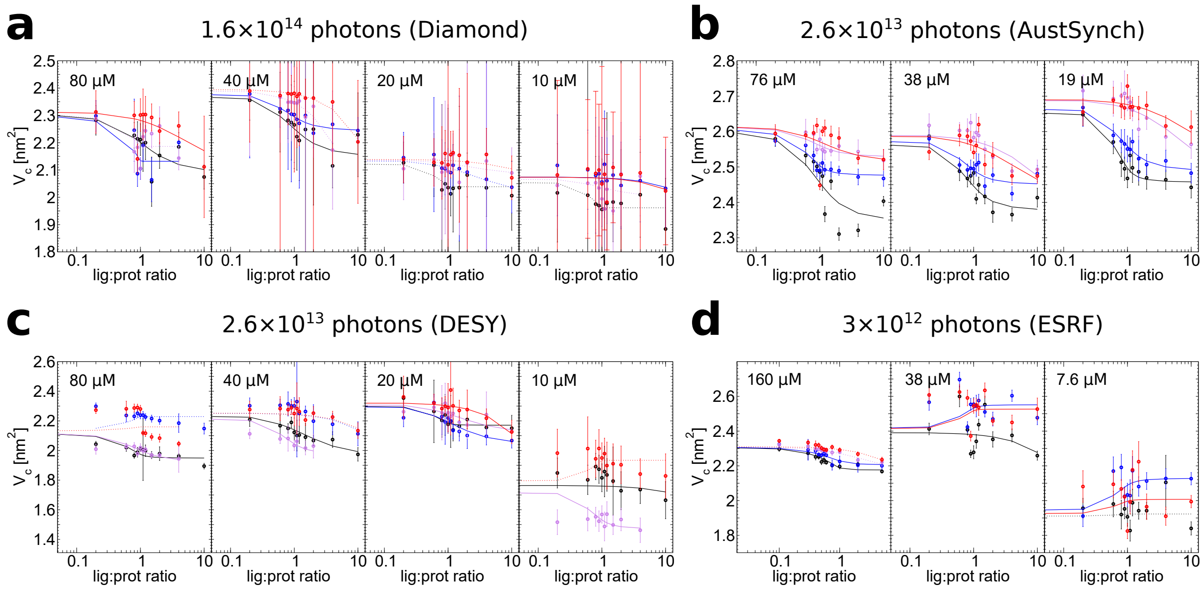

Figure S7. Replicate V_c titration curves of HisBP against four ligands His (black), Arg (blue), Lys (violet), and Orn (red), at Diamond (a), DESY (b), Australian Synchrotron (c), and ESRF (d). The HisBP concentrations used within each experiment are labelled on respective plots.

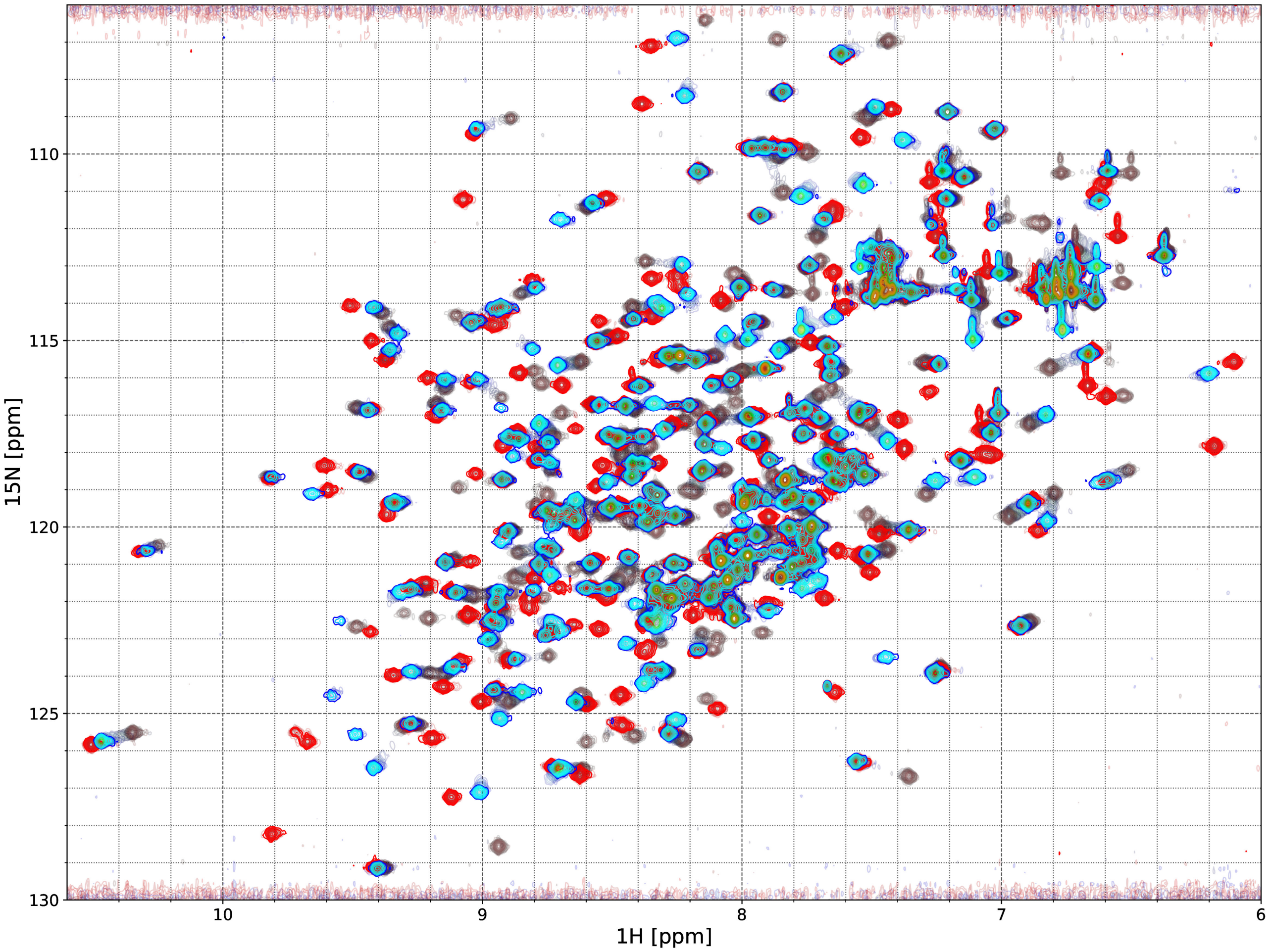

Figure S8. Titration of HisBP versus Histidine (red) and Arginine (blue-teal) by NMR, depicted by overlaying HSQC plots at multiple ligand:protein ratios between 0.0∼1.5. Resonance peaks of apo-HisBP are shown in grey, while resonance peaks from intermediate titration points are shown in light grey with gradually increasing color intensity until color saturation is reached at a ratio of 1.5:1.0. Partial differences in peak-shifts between HisBP:His and HisBP:Arg titrations indicate subtle differences in the final bound conformation.

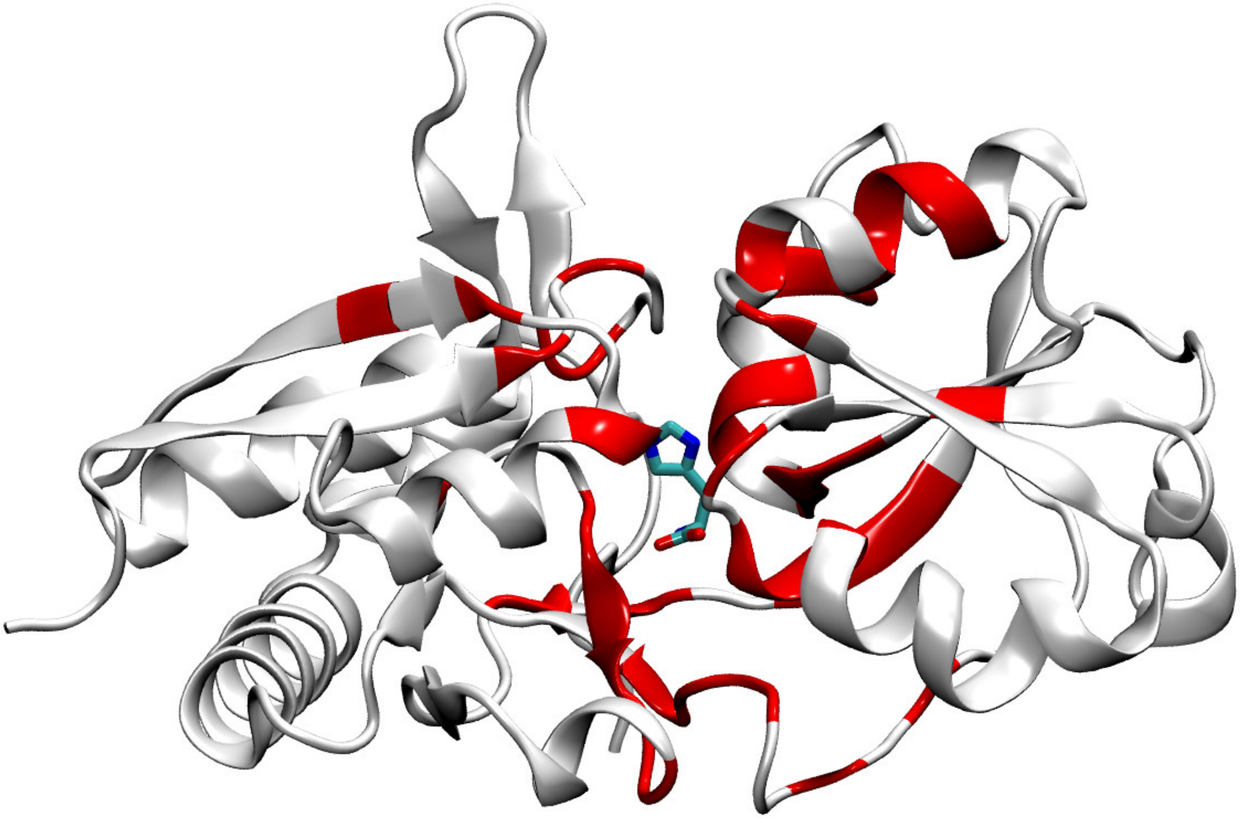

Figure S9. Depiction of HisBP crystal structure bound to Histidine (1HSL.pdb chain A), shown as white cartoons and sticks, respectively. Residues have been colored in red, where the ^{15}N-^{1}H HSQC peaks of His-bound HisBP do not exhibit any overlap with peaks found in Arg-bound HisBP. Concentrations around the active-site cleft and inter-domain hinge suggest minor but extensive rearrangements to accommodate the larger Arg sidechain.

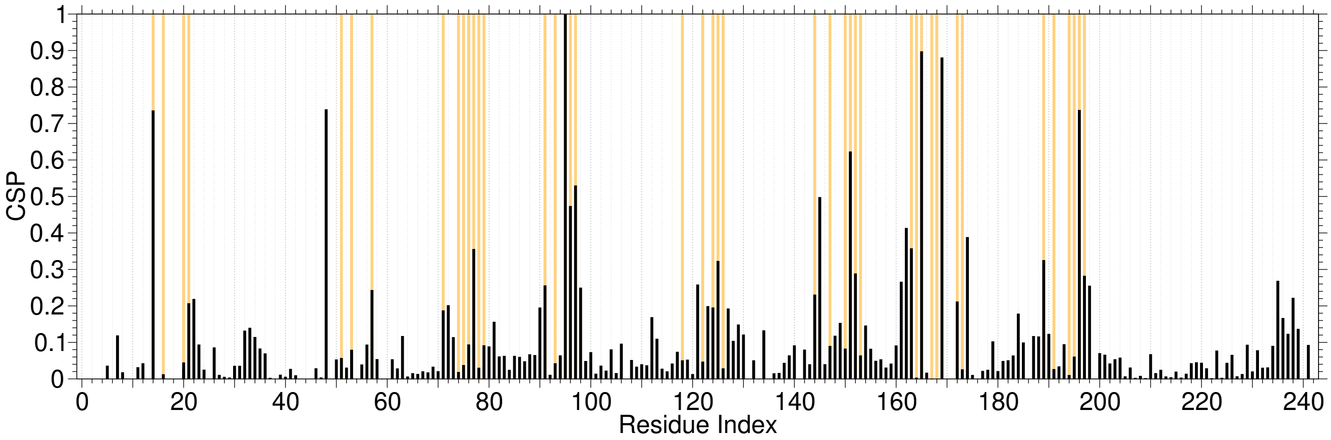

Figure S10. Residue-wise chemical-shift perturbations for Arg-bound HisBP relative to apo-HisBP (black). For comparison, residues highlighted in Figure S9 are shown here in orange.

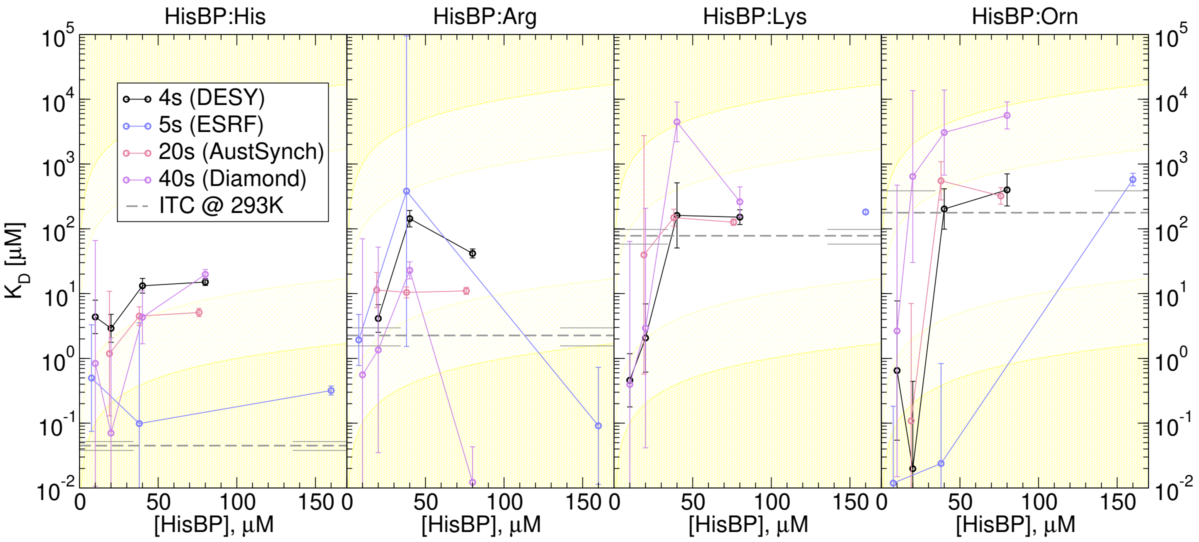

Figure S11. HisBP screens replicated at DESY P12 (black), Australian Synchrotron SAXS/WAXS (red), Diamond B21 (violet) and ESRF BM29 (blue), using $\chi_{\mathrm{lin.}}$ to derive relative populations. The affinity K_D of HisBP versus four ligands is evaluated at different protein concentrations to test prediction capability versus ITC values (dotted grey with error bars). Regions where K_D predictions lie outside 1 and 2 orders of magnitude of input protein concentrations are shaded in light and dark yellow, representing regions of decreased confidence.

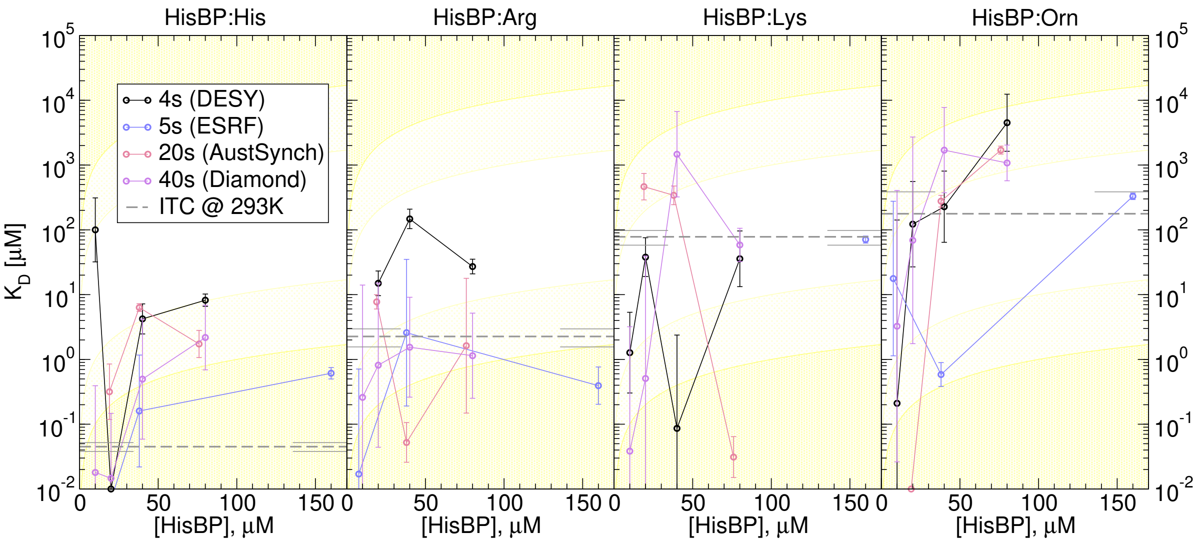

Figure S12. HisBP screens replicated at DESY P12 (black), Australian Synchrotron SAXS/WAXS (red), Diamond B21 (violet) and ESRF BM29 (blue), using R_{g} to derive relative populations. The affinity K_D of HisBP versus four ligands is evaluated at different protein concentrations to test prediction capability versus ITC values (dotted grey with error bars). Regions where K_D predictions lie outside 1 and 2 orders of magnitude of input protein concentrations are shaded in light and dark yellow, representing regions of decreased confidence.

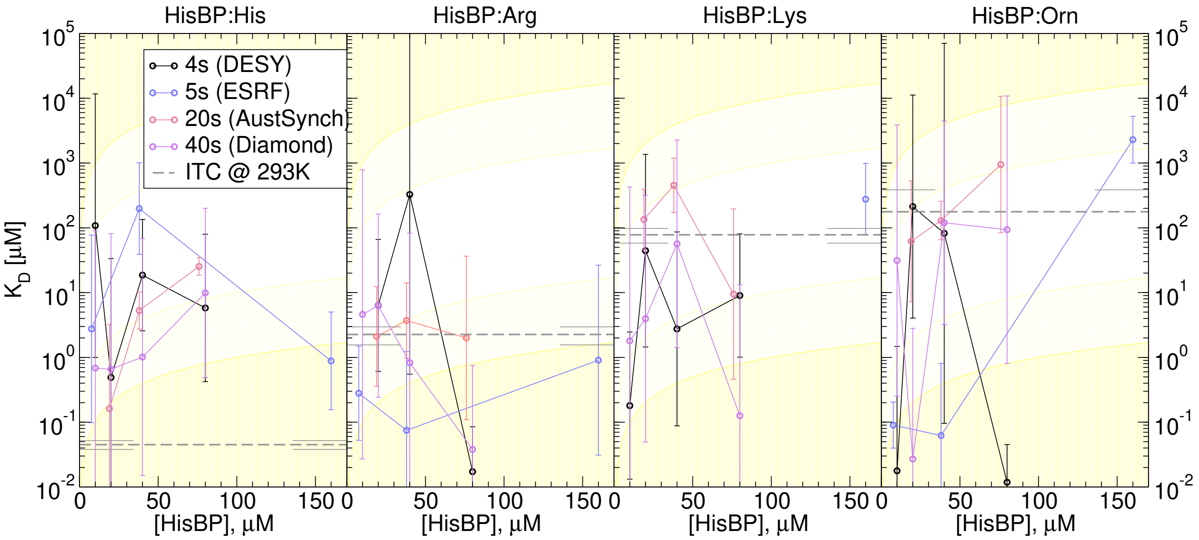

Figure S13. HisBP screens replicated at DESY P12 (black), Australian Synchrotron SAXS/WAXS (red), Diamond B21 (violet) and ESRF BM29 (blue), using V_c to derive relative populations. The affinity K_D of HisBP versus four ligands is evaluated at different protein concentrations to test prediction capability versus ITC values (dotted grey with error bars). Regions where K_D predictions lie outside 1 and 2 orders of magnitude of input protein concentrations are shaded in light and dark yellow, representing regions of decreased confidence.

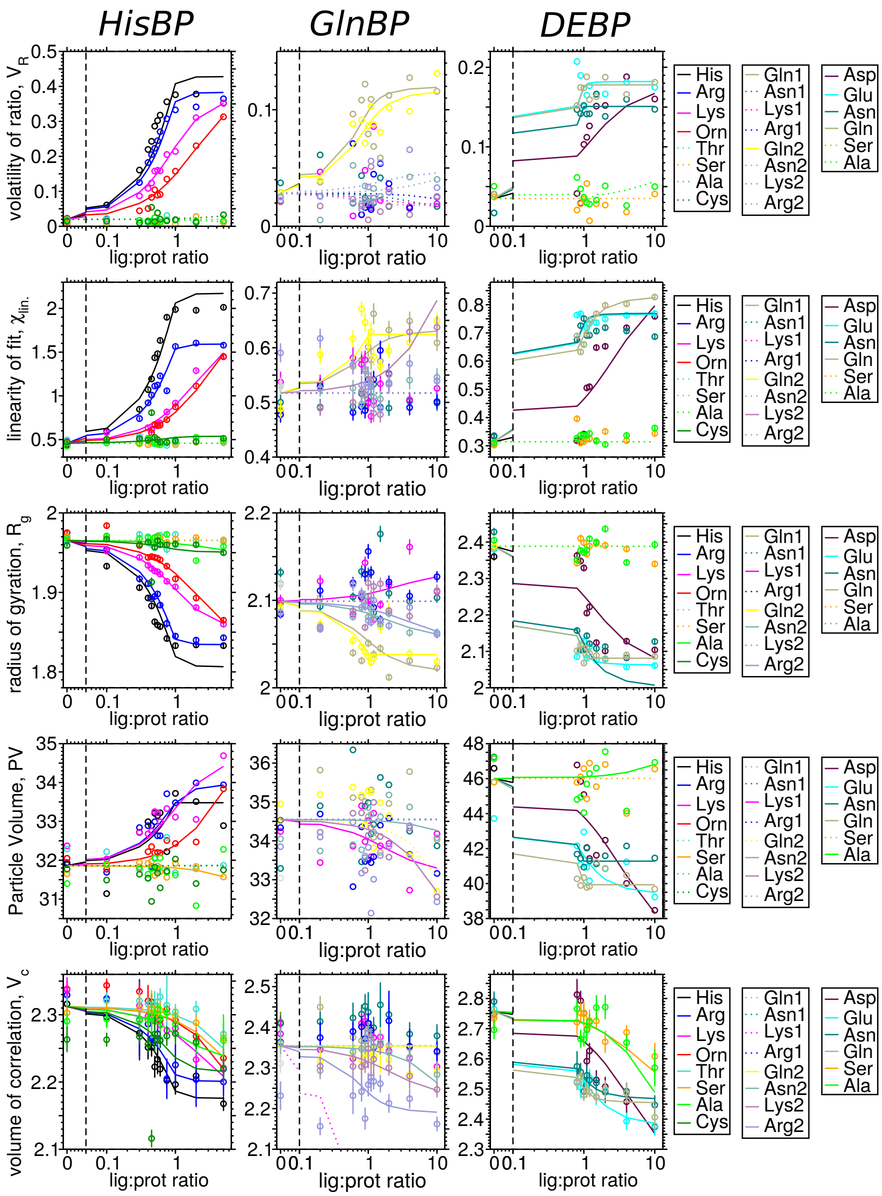

Figure S14. Titration of three periplasmic binding proteins at ESRF BM29 SAXS HisBP (left), GlnBP(center), and DEBP(right). Measured SAXS perturbations are expressed in a number of structural and scattering parameters, some of which form clear ligand-dependence. From top: volatility of ratio V_{R}, linearity of fit $\chi_{\mathrm{lin.}}$, radius of gyration R_{g}, particle volume, and volume of correlation V_{c}.

Supplementary Tables

Protein	Method	exposure	conc. (μM)	Ligand identity and dissociation constant K_D			
				His	Arg	Lys	Orn
HisBP	V_R (DESY)	4 s	80	6.4±1.1 μM	5.9±0.6 μM	58±7.5 μM	100± 26 μM
	V_R (DESY)	4 s	40	3.7±0.58 μM	74± 19 μM	720±350 μM	120± 43 μM
	V_R (DESY)	4 s	20	1±0.19 μM	33±4.5 μM	61±6.4 μM	130±170 μM
	V_R (DESY)	4 s	10	84± 68 μM	n.d.	2.3±1.6 nM	130±2500 μM
	V_R (ESRF)	5 s	160	370±470 nM	710±1000 nM	49±4.1 μM	250± 24 μM
	V_R (ESRF)	5 s	38	240±140 nM	2±1.9 μM	n.d.	5.4± 28 mM
	V_R (ESRF)	5 s	7.6	51±2900 μM	2±190 μM	n.d.	38± 53 μM
	V_R (AustSynch)	20 s	76	3.6±0.74 μM	5.5±1.4 μM	95± 11 μM	220± 34 μM
	V_R (AustSynch)	20 s	38	1.5±0.7 μM	3±1.4 μM	32±4.6 μM	190± 39 μM
	V_R (AustSynch)	20 s	19	62± 28 nM	2.8±0.31 μM	110± 10 μM	130± 24 μM
	V_R (Diamond)	40 s	80	1.3±0.5 μM	7.3±1.9 μM	89±9.6 μM	640±160 μM
	V_R (Diamond)	40 s	40	13± 11 nM	4.8±0.48 μM	320±210 μM	680±360 μM
	V_R (Diamond)	40 s	20	5.1±4.1 nM	920±110 nM	47±3.6 μM	1.4±1.1 mM
	V_R (Diamond)	40 s	10	1.4±0.92 nM	370±140 nM	230±200 μM	1.2±1.8 mM

Table S1. Raw fitted dissociation constant K_D to SAXS V_R curves for all HisBP titrations. Noting that values outside of ~2 orders of magnitudes of HisBP concentration are unreliable.

Protein	Method	exposure	conc. (μM)	Ligand identity and dissociation constant K_D			
				His	Arg	Lys	Orn
HisBP	$\chi_{lin.}$ (DESY)	4 s	80	15±1.9 μM	42±6.7 μM	150± 40 μM	400±240 μM
	$\chi_{lin.}$ (DESY)	4 s	40	13±3.5 μM	140± 42 μM	160±230 μM	200±160 μM
	$\chi_{lin.}$ (DESY)	4 s	20	2.9±1.5 μM	4.1±2.1 μM	2.1±3.2 μM	20±220 nM
	$\chi_{lin.}$ (DESY)	4 s	10	4.4±2.8 μM	n.d.	460±500 nM	650±3800 nM
	$\chi_{lin.}$ (ESRF)	5 s	152	320± 51 nM	91±360 nM	180± 15 μM	580±130 μM
	$\chi_{lin.}$ (ESRF)	5 s	38	99±1800 nM	380±47000 μM	n.d.	24±420 nM
	$\chi_{lin.}$ (ESRF)	5 s	7.6	500±1600 nM	1.9± 2 μM	n.d.	12± 91 nM
	$\chi_{lin.}$ (AustSynch)	20 s	76	5.1±0.75 μM	11±1.4 μM	130± 14 μM	320± 96 μM
	$\chi_{lin.}$ (AustSynch)	20 s	38	4.5±1.5 μM	10± 2 μM	150± 46 μM	550±400 μM
	$\chi_{lin.}$ (AustSynch)	20 s	19	1.2±5.4 μM	11±7.6 μM	40±1400 μM	110±3500 nM
	$\chi_{lin.}$ (Diamond)	40 s	80	20±3.4 μM	12± 20 nM	260±150 μM	5.6±2.8 mM
	$\chi_{lin.}$ (Diamond)	40 s	40	4.3±4.7 μM	23±7.1 μM	4.5±3.4 mM	3.1±6.6 mM
	$\chi_{lin.}$ (Diamond)	40 s	20	70±1000 nM	1.4± 26 μM	2.9±100 μM	640±6800 μM
	$\chi_{lin.}$ (Diamond)	40 s	10	840±33000 nM	560±35000 nM	400±32000 nM	2.7±240 μM

Table S2. Raw fitted dissociation constant K_D to SAXS $\chi_{lin.}$ curves for all HisBP titrations. Noting that values outside of ~2 orders of magnitudes of HisBP concentration are unreliable.

Protein	Method	exposure	conc. (μM)	Ligand identity and dissociation constant K_D			
				His	Arg	Lys	Orn
HisBP	R_g (DESY)	4 s	80	8.2±1.8 μM	27±7.1 μM	36± 42 μM	4.5±5.4 mM
	R_g (DESY)	4 s	40	4.2±2.4 μM	150± 52 μM	86±1200 nM	230±370 μM
	R_g (DESY)	4 s	20	2.7±4.7 nM	15±6.8 μM	38± 28 μM	120±270 μM
	R_g (DESY)	4 s	10	100±140 μM	n.d.	1.3±2.5 μM	210±71000 nM
	R_g (ESRF)	5 s	160	610±120 nM	390±280 nM	71±8.9 μM	330± 38 μM
	R_g (ESRF)	5 s	38	160±570 nM	2.6± 17 μM	n.d.	580±250 nM
	R_g (ESRF)	5 s	7.6	0.76±2.1e-05 nM	17±360 nM	n.d.	18±140 μM
	R_g (AustSynch)	20 s	76	1.7±0.87 μM	1.6±8.8 μM	31± 25 nM	1.7±0.24 mM
	R_g (AustSynch)	20 s	38	6.3±0.87 μM	52± 40 nM	340±120 μM	280± 58 μM
	R_g (AustSynch)	20 s	19	320±370 nM	7.7± 2 μM	460±230 μM	2.3±3.9 nM
	R_g (Diamond)	40 s	80	2.2±3.1 μM	1.1±2.5 μM	58± 37 μM	1.1±0.73 mM
	R_g (Diamond)	40 s	40	500±2100 nM	1.5±4.4 μM	1.5±3.2 mM	1.7±3.7 mM
	R_g (Diamond)	40 s	20	15± 73 nM	810±7500 nM	510±20000 nM	69±1400 μM
	R_g (Diamond)	40 s	10	18±200 nM	260±7000 nM	38±1600 nM	3.3±200 μM

Table S3. Raw fitted dissociation constant K_D to SAXS R_g curves for all HisBP titrations. Noting that values outside of ~2 orders of magnitudes of HisBP concentration are unreliable.

Protein	Method	exposure	conc. (μM)	Ligand identity and dissociation constant K_D			
				His	Arg	Lys	Orn
HisBP	V_c (DESY)	4 s	80	5.8± 40 μM	17± 41 nM	9± 40 μM	12± 21 nM
	V_c (DESY)	4 s	40	19± 66 μM	330±97000 μM	2.8± 43 μM	82±35000 μM
	V_c (DESY)	4 s	20	490±17000 nM	6.3± 33 μM	44±680 μM	210±5600 μM
	V_c (DESY)	4 s	10	110±5900 μM	n.d.	180±1200 nM	18±740 nM
	V_c (ESRF)	5 s	160	880±2400 nM	910±13000 nM	280±450 μM	2.3±2.1 mM
	V_c (ESRF)	5 s	38	200±490 μM	75±610 nM	n.d.	62±400 nM
	V_c (ESRF)	5 s	7.6	2.8± 39 μM	280±720 nM	n.d.	90± 82 nM
	V_c (AustSynch)	20 s	76	25±8.1 μM	2± 18 μM	9.5± 98 μM	950±5300 μM
	V_c (AustSynch)	20 s	38	5.3±3.7 μM	3.7±6.6 μM	450±510 μM	130± 96 μM
	V_c (AustSynch)	20 s	19	160±1600 nM	2.1± 6 μM	130±170 μM	62±260 μM
	V_c (Diamond)	40 s	80	9.9±100 μM	38±380 nM	130±6600 nM	94±5400 μM
	V_c (Diamond)	40 s	40	1± 34 μM	830±41000 nM	57±1100 μM	120±2200 μM
	V_c (Diamond)	40 s	20	660±41000 nM	6.3± 81 μM	3.9±160 μM	27±1400 nM
	V_c (Diamond)	40 s	10	680±45000 nM	4.6±390 μM	1.8±210 μM	31±1900 μM

Table S4. Raw fitted dissociation constant K_D to SAXS V_c curves for all HisBP titrations. Noting that values outside of ~2 orders of magnitudes of HisBP concentration are unreliable.

Table S5. Detailed reporting for SAXS measurements for six samples, measured at ESRF BM29 during Sep. 2018: (i) HisBP in free state and bound to ten-fold molar excess His, (ii) GlnBP in free state and bound to ten-fold molar excess Gln, amd (iii) DEBP in free state and bound to ten-fold molar excess Glu.

	$HisBP_{free}$	$HisBP_{bound}$	$GlnBP_{free}$	$GlnBP_{bound}$	$DEBP_{free}$	$DEBP_{bound}$
(a) Sample Details						
Source organism	*E. coli*					
Expression organism	*E. coli* BL21(DE3)					
Plasmid source	this work		Prof. Colin Jackson			
Description	P0AEU0 (23-260) with N-terminal Gly		P0AEQ3 (23-248) with N-terminal His_6		P37902 (28-302) with N-terminal His_6	
Computed extinction coefficient ϵ_{280nm} (M^{-1} cm^{-1})	17,545		25,900		24,075	
Molecular mass M from chemical composition (kDa)	26.290		25.786		32.052	
loading concentration (mg ml^{-1})	4.2		2.0		2.2	
injection volume (μl)	50		50		50	
Concentration (μM)	160		80		70	
Solvent composition and source	100 mM NaCl, 20 mM $NaPO_4$, 0.5 mM TCEP, pH 7.4					
(b) SAS data collection parameters						
Source and instrument	Grenoble ESRF BM29 with Dectris Pilatus 1M					
Wavelength (Å)	0.9919					
Sample-detector distance (m)	2.849					
q-measurement range (nm^{-1})	0.0306–4.9462					
Intensity Normalization	0.00192					
Radiation damage monitoring	frame-by-frame comparison					
Exposure time (s) & number	2.0×12 frames					
Sample configuration	96-well plate with flow-through capillary measurement					
Sample temperature (°C)	20					
(c) Software employed for SAS data reduction, analysis and interpretation						
SAXS data processing	SAXScreen and ATSAS 2.8					
Molecular graphics	Visual Molecular Dynamics					
(d) Structural parameters						
Guinier analysis (PRIMUS)						
$I(0)$ (raw)	124.42±0.10	128.71±0.10	49.40±0.08	49.65±0.06	67.04±0.11	67.06±0.08
R_g (nm)	1.96±0.07	1.83 ±0.10	2.11±0.14	2.03±0.13	2.32±0.21	2.06±0.13
q-range (nm^{-1})	0.1249–0.6580	0.0825–0.7052	0.0919–0.6156	0.0966–0.6391	0.0872–0.5495	0.1297–0.6297
Coefficient of correl. R^2	0.97	0.99	0.94	0.95	0.96	0.95
M from $I(0)$ (kDa, ratio to expected value)	29.6 (1.13)	n.a.	24.7 (0.96)	n.a.	30.47 (0.95)	n.a.
$P(r)$ Analysis (AUTOGNOM)						
$I(0)$ (cm^{-1})	124.7±0.1	128.8±0.1	49.13±0.09	49.47±0.09	67.28±0.12	66.97±0.10
R_g (nm)	1.975±0.002	1.833±0.002	2.097±0.005	2.023±0.004	2.380±0.008	2.061±0.005
d_{max} (nm)	6.03	5.71	6.18	6.10	8.46	6.44
q-range (nm^{-1})	0.1014–2.7998					
GNOM total est.	0.9759	0.9691	0.8962	0.8913	0.7415	0.8793
M from $I(0)$ (ratio to expected value)	29.7 (1.13)	n.a.	24.6 (0.95)	n.a.	30.6 (0.95)	n.a.
(g) Data and model deposition IDs						
SASBDB	SASDFD8	SASDFE8	SASDFF8	SASDFG8	SASDFH8	SASDFJ8
